# Electron-lattice interactions strongly renormalize the charge-transfer energy in the spin-chain cuprate Li_2_CuO_2_

**DOI:** 10.1038/ncomms10563

**Published:** 2016-02-17

**Authors:** Steve Johnston, Claude Monney, Valentina Bisogni, Ke-Jin Zhou, Roberto Kraus, Günter Behr, Vladimir N. Strocov, Jiři Málek, Stefan-Ludwig Drechsler, Jochen Geck, Thorsten Schmitt, Jeroen van den Brink

**Affiliations:** 1 Department of Physics and Astronomy, The University of Tennessee, Knoxville, Tennessee 37996, USA; 2 Research Department Synchrotron Radiation and Nanotechnology, Paul Scherrer Institut, CH-5232, Villigen, Switzerland; 3 Department of Physics, University of Zurich, Winterthurerstrasse 190, CH-8057 Zurich, Switzerland; 4 Leibniz Institute for Solid State and Materials Research, IFW Dresden, Helmholtzstrasse 20, D-01171 Dresden, Germany; 5 National Synchrotron Light Source II, Brookhaven National Laboratory, Upton, New York 11973-5000, USA; 6 Diamond Light Source, Harwell Science and Innovation Campus, Didcot, Oxfordshire OX11 0DE, UK; 7 Institute of Physics, ASCR, Na Slovance 2, CZ-18221 Praha 8, Czech Republic; 8 Department of Physics, TU Dresden, D-01062 Dresden, Germany

## Abstract

Strongly correlated insulators are broadly divided into two classes: Mott–Hubbard insulators, where the insulating gap is driven by the Coulomb repulsion *U* on the transition-metal cation, and charge-transfer insulators, where the gap is driven by the charge-transfer energy Δ between the cation and the ligand anions. The relative magnitudes of *U* and Δ determine which class a material belongs to, and subsequently the nature of its low-energy excitations. These energy scales are typically understood through the local chemistry of the active ions. Here we show that the situation is more complex in the low-dimensional charge-transfer insulator Li_2_CuO_2_, where Δ has a large non-electronic component. Combining resonant inelastic X-ray scattering with detailed modelling, we determine how the elementary lattice, charge, spin and orbital excitations are entangled in this material. This results in a large lattice-driven renormalization of Δ, which significantly reshapes the fundamental electronic properties of Li_2_CuO_2_.

## Abstract (web summary)

In transition metal oxides, an insulating band gap is found when the energy scales related to ionic charge excitations dominate over electronic itinerancy. Here, the authors demonstrate strong electron-phonon interactions in Li_2_CuO_2_ and their effect on the insulating band gap.

## Graphical abstract

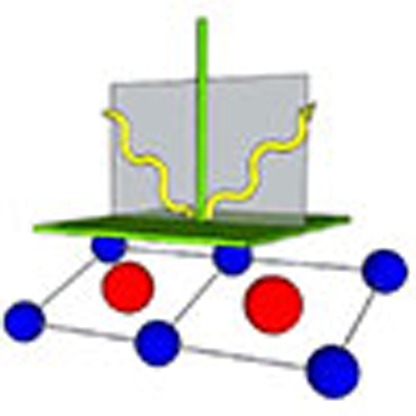

The celebrated Zaanen–Sawatzky–Allen classification scheme^1^ divides strongly correlated insulators, such as transition-metal oxides (TMOs), into two broad categories: charge-transfer (CT) or Mott–Hubbard insulators. Two fundamental energy scales determine the boundary between these categories. The first is the Coulomb repulsion *U* associated with the transition-metal cation site, which parameterizes the energy cost for (*d*^*n*−1^*d*^*n*+1^)-type charge excitations. The second is the CT energy Δ associated with (d^*n*−1^L)-type charge excitations, where a hole moves from the cation site to the ligand anions L. When these atomic energy scales dominate over electron itinerancy, the emerging insulator is of the CT type when Δ<*U* and of the Mott–Hubbard type when Δ>*U* (ref. 1).

Determining which factors set the magnitude of these scales is important for the most basic understanding of the behaviour of TMOs. In an ionic picture, the on-site Coulomb interaction *U* sets the splitting of the lower and upper Hubbard bands^1^,^2^, while the CT energy 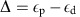 is typically set by the relative electronegativity of the oxygen (O) anions and the ionization energy of the transition-metal cation^2^. As such, copper oxides are typically classified as CT insulators, where their conduction band is derived from the copper (Cu) states forming the upper Hubbard band, while the valence band is derived from the O 2*p* states. This dichotomy creates a fundamental asymmetry between electron and hole doping processes, as reflected for example in the phase diagram of the high-temperature superconducting cuprates^3^,^4^.

Properly classifying a real material is a challenging task experimentally. One needs to be able to determine the size of Δ and *U* in the presence of complications such as hybridization effects and additional interactions. Resonant inelastic X-ray scattering (RIXS) is a powerful spectroscopic tool in this context^5^,^6^. It is capable of directly probing charge^7^,^8^,^9^,^10^, orbital^11^, spin^12^,^13^,^14^,^15^ and, as most recently discovered, lattice excitations^16^,^17^,^18^,^19^. The observation of the latter is particularly exciting, as RIXS can access the electron–phonon (e-ph) coupling strength directly^17^, and with element specificity^18^. This opens a direct means to study the influence of lattice dynamics on the fundamental electronic energy scales.

In this work we perform such a study for the edge-shared CT insulator Li_2_CuO_2_ (LCO) to determine how the e-ph interaction helps to shape the CT energy in this quasi-one-dimensional spin-chain cuprate. The active electronic degrees of freedom in LCO are formed from edge-shared CuO_4_ plaquettes with a central Cu 3*d*^9^ cation^20^,^21^,^22^. As a result, LCO harbours Zhang-Rice singlet (ZRS) charge excitons similar to those found in the high-*T*_c_ cuprates^8^,^9^,^23^. The e-ph interaction is also expected to play a role in this system. This was recently demonstrated for the related edge-shared cuprate Ca_2+x_Y_2−x_Cu_5_O_10_ (CYCO), where charge carriers couple strongly to Cu–O bond-stretching phonon modes polarized perpendicular to the chain direction^18^,^19^. We demonstrate here that a similar e-ph interaction occurs in LCO. More importantly, however, we show that this interaction provides a substantial contribution to Δ, accounting for ≈54% of its total value. This result is obtained from a comprehensive analysis of high-resolution oxygen K-edge RIXS^5^,^6^ data that resolves individual phonon, *dd*, and CT excitations (including the ZRS exciton). This in turn allows us to disentangle the elementary spin, charge, orbital and lattice excitations over an energy range of ∼10 eV. If the e-ph interaction is omitted in our analysis, the spectra imply a value Δ≈4.6 eV; however, when the e-ph interaction is properly accounted for, this value separates into a purely electronic contribution of Δ_el_≈2.1 eV, and a very substantial phononic contribution of about the same size Δ_ph_≈2.5 eV. As such, the elementary excitations across the CT gap in LCO couple strongly to specific phonon modes, enhancing their total energy cost. This result places the basic classification of LCO in a new light, where the relevant energy scales are shaped not only by the local chemistry of the atoms but also dynamically by interactions with phonons that are relevant for many TMOs^24^,^25^,^26^,^27^.

## Results

### RIXS at the oxygen K-edge in LCO

**Figure 1:**
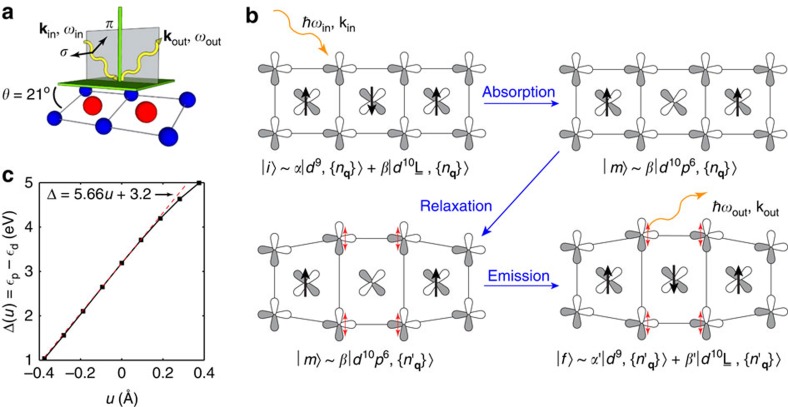
A cartoon sketch of the RIXS process. (**a**) A sketch of the experimental scattering geometry, showing the scattering plane (grey plane) perpendicular to the sample surface (green plane), making an angle of 21° with the CuO_2_ chains, depicted here as a Cu_2_O_6_ dimer (Cu in red and O in blue). The wavy lines represent the incoming and outgoing photons while the black arrows indicate the polarization of the incoming photons with respect to the scattering plane. (**b**) A sketch of the RIXS excitation process whereby the lattice is excited. The initial electronic state is predominantly of |*i*〉_el_∼*α*|*d*^9^〉+|*d*^10^L〉 character, where L denotes a hole delocalized on the ligand-oxygen sites, while the initial lattice state involves a coherent state of phonon quanta 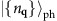 describing the shifted equilibrium position of the O atoms. The thick black arrows represent the spins of the Cu 3d holes in the LCO chain. After the 1*s*→2*p* transition, an intermediate state of 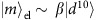 character is formed, corresponding to an upper Hubbard band excitation where the number of holes on the Cu site has changed. Following this, the lattice relaxes in response to the change in Cu density, until the 1*s* core hole is filled, leaving the system in an excited electronic and lattice configuration 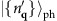. The red arrows indicate the direction of the O atom's motion. (**c**) The variation of the CT energy 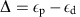 as a function of a static compression *u* of the Cu–O chains in a direction perpendicular to the chain direction. Crystal field effects have been neglected. The black points are the calculation results while the red dashed line is a linear fit to these data at small displacement.

The oxygen K-edge RIXS process is sketched in Fig. 1. During the experiment, photons with energy *ℏω*_in_ and momentum *ℏ***k**_in_ are absorbed by the system in its initial state 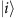 via an O 1*s*→2*p* dipole transition. This creates an intermediate state 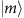 with an O 1*s* core hole and an additional electron in the conduction band. The resulting intermediate state then evolves in time under the influence of the core-hole potential and the excited electronic configuration. A number of elementary excitations are created in the system during this time until the core-hole decays, emitting an outgoing photon (momentum *ℏ***k**_out_ and energy *ℏω*_out_) and leaving the system in an excited final state |*f*〉.

To understand how the e-ph interaction enters this process it is important to examine further the states involved. The electronic ground state in LCO, and other spin-chain cuprates, is largely of |*i*_el_〉∼*α*|*d*^9^〉+*β|d*^10^L〉 character, where L denotes a hole on the ligand O. This state, however, couples strongly to Cu–O bond-stretching phonons like the transverse mode sketched in Fig. 1b. This coupling can occur in two ways. For instance, the bond-stretching modes directly modulate the Cu–O hopping integral. Alternatively, these modes can modulate the Madelung energy of the central Cu atom, effectively lowering/raising the energy of the Cu site as the O atoms move closer to/further from it. This latter mechanism cannot be effectively screened in lower dimensions, and turns out to be the relevant coupling mechanism for our analysis^24^,^28^. Since the electronic contribution to the CT energy (in hole language) in this system is 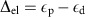, we can view the phonon modes as modulating the CT energy^18^. This is confirmed in Fig. 1c, where we plot the linear variation in Δ_el_ obtained from a static point charge model under uniform expansions/compressions of the CuO plaquettes in the direction perpendicular to the chain (Methods section).

The physical interpretation of this result is as follows. The lighter O atoms, in an effort to eliminate the first-order e-ph coupling and minimize the energy of the system, shift to new equilibrium positions located closer towards the Cu atoms. Subsequently, the new ground state of the system involves a coherent state of phonon quanta {*n*_*q*_} that describes the distorted structure. The new equilibrium positions also produce changes in the Madelung energy of the Cu site, increasing the CT energy in comparison to the value obtained in the absence of the interaction. This renormalization of the CT energy is a bulk property of the crystal arising from the e-ph interaction with the Cu 3*d*^9^ hole present in the ground state. As such, it will manifest in many bulk spectroscopies including RIXS (this work), optical conductivity (Supplementary Fig. 1), and inelastic neutron scattering (Supplementary Note 1). It is important to note, however, that this renormalization is inherently dynamic, as the oxygen atoms are free to respond to changes in Cu hole density. This has observable consequences in the RIXS spectra, as we now demonstrate.

The RIXS process for LCO's initial state dressed by the phonon excitations is sketched in Fig. 1b. At low temperatures it is now predominantly |*i*〉∼*α*|*d*^9^,{*n*_*q*_}〉+*β*|*d*^10^*L*,{*n*_**q**_}〉 in character. The intermediate state is formed after the creation of a core hole on the O site, through an O 1*s*→2*p* transition. This creates an intermediate state of |*m*〉∼*β*|*d*^10^*p*^6^,{*n*_**q**_}〉 character, which corresponds to an upper Hubbard band excitation, where the number of holes on the Cu site has changed. In response, the ligand O atoms begin to relax towards new positions until the core-hole decays. Ultimately, this leaves the system in a final state with both excited electronic and lattice configurations 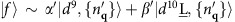.

It is important to stress that here the core-hole provides us with a lens through which we can view the e-ph interaction using RIXS. The core-hole does not create the interaction. While the lattice excitations we probe are being generated in the intermediate state, they carry information about the strength of the e-ph interaction that is present in the initial and final states. The change in carrier density introduced by the creation of the core hole excites the lattice, but the way in which the lattice responds depends on strength and details of the interaction.

### Electron–phonon coupling in the RIXS data

**Figure 2:**
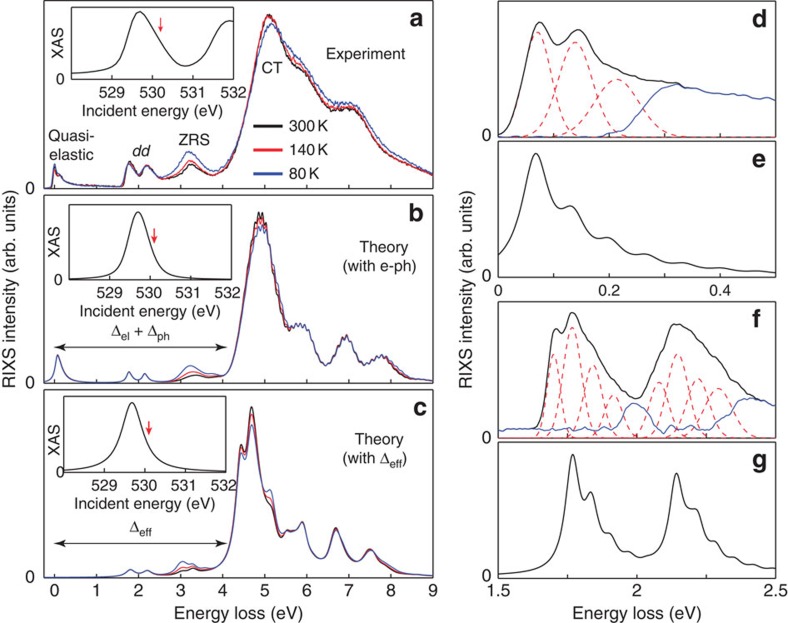
XAS and RIXS spectra of LCO at the oxygen *K*-edge. (**a**) The measured RIXS spectra, recorded at various temperatures, as indicated. The incident photon energy for these measurements was detuned slightly from the upper Hubbard band peak in the XAS, as shown in the inset. The incident phonon energy is indicated by the red arrow. (**b**) The calculated RIXS spectra obtained using a cluster model that includes coupling to the O–O bond-stretching mode. The calculated XAS spectrum is shown in the inset. For comparison, **c** shows calculated spectra obtained from a model without coupling to the phonon mode and with an increased value of 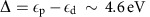. The detailed measured RIXS spectra highlighting the harmonic phonon excitations in the quasi-elastic and *dd*-excitation energy loss range are shown in **d** and **f**, respectively. Here, the red dashed lines show Gaussian fits to these data that highlight the individual phonon excitations. The blue line is the difference between the data and the red dashed lines. The corresponding RIXS calculations are shown in **e** and **g**, respectively. In **d**–**g** the incident photon energy coincides with the peak in the XAS intensity. Note that the elastic line has been removed from all of the calculated RIXS spectra for clarity. The spectra in **e** and **g** have been broadened using a Gaussian line shape with a s.d. of 60 meV. In **b** and **c** this width was increased to 130 meV to mimic additional broadening of CT features due to the bands formed by the O 2*p* orbitals that are not well captured by our small Cu_3_O_8_ cluster calculation.

The presence of the e-ph interaction in LCO is confirmed in our measured RIXS spectra, shown in Fig. 2a. The X-ray absorption spectroscopy (XAS) spectrum (inset) has a prominent peak centred at 529.7 eV, which corresponds to the discussed excitation into the upper Hubbard band. The RIXS spectra, taken with incident photons detuned slightly from this energy (*ℏω*_in_=530.08 eV, indicated by the arrow), are rich. (Here we have shown data detuned from the UHB resonance since the intensity of the ZRS excitation is largest for this incident photon energy^9^.) We observe a number of features, including a long tail of intensity extending from the elastic line comprised of several phonon excitations; two nearly T-independent peaks at ∼1.7 and ∼2.1 eV, which correspond to now well-known *dd* excitations^23^,^29^; a T-dependent peak at ∼3.2 eV, which corresponds to a ZRS excitation^8^,^9^; and, finally, a band of CT excitations for *ℏ*Ω=*ℏω*_out_−*ℏω*_in_>4. Here, we are using the term CT excitation as an umbrella term for any excitation where a Cu 3*d* hole has been transferred to the O 2*p* orbitals, with the exception of the ZRS excitation. As such, CT excitations include the fluorescence excitations. We have explicitly confirmed each of these identifications by examining the character of the final state wave functions obtained from our model calculations.

The phonon excitations are more apparent in the high-resolution measurements of the quasi-elastic and *dd*-excitation energy range, shown in Fig. 2d,f, respectively. We observe clear harmonic phonon excitations separated in energy by *ℏ*Ω_ph_∼74 meV, consistent with those reported for CYCO^18^,^19^. This demonstrates that the e-ph coupling is a common phenomenon in the spin-chain cuprates. Another important aspect of the data is the positions of the ZRS and CT excitations, which are determined by the CT energy. From these data we infer Δ∼4.6 eV, which is significantly >3.2 eV obtained from Madelung energy estimates based solely on local chemistry considerations^22^. This discrepancy can be accounted for by including the bond-stretching phonons implied by the observed harmonic excitations in Fig. 2d,f.

### Electron–phonon contribution to the CT energy

We assessed the phonon contribution to Δ by modelling the RIXS spectra within the Kramers–Heisenberg formalism^5^,^6^. The initial, intermediate and final states were obtained from small cluster exact diagonalization calculations that included the lattice degrees of freedom^9^,^18^. The electronic model and its parameters are the same as those used in a previous LCO study^9^, however, we have extended this model to include additional Cu 3*d* orbitals and kept the bare CT energy 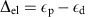 as a fitting parameter. This number represents the size of the CT energy in the absence of the e-ph interaction. The model for the lattice degrees of freedom is similar to ref. 18 but with an e-ph coupling strength parameterized by *g* and the phonon energy *ℏ*Ω_ph_=74 meV, as determined from our data (Methods section). The calculated spectra are shown in Fig. 2b,e,g, where we have set Δ_el_=2.14 eV and *g*=0.2 eV. This choice produces the best global agreement between the theory and experiment both in terms of the positions of the CT and ZRS excitations, as well as the intensities of the harmonic excitations in the *dd* and quasi-elastic regions. We also stress that the remaining parameters of the model were held fixed during our fitting procedure, as their values are heavily constrained by optical conductivity^30^,^31^, electron energy-loss spectroscopy (EELS)^31^, inelastic neutron scattering^32^ and RIXS (this work and ref. 9) measurements.

A closer inspection of Fig. 2b reveals our main finding: the observed positions of the ZRS and CT excitations are not set by the purely electronic value of Δ_el_=2.14 eV but rather the total Δ=Δ_el_+Δ_ph_∼4.6 eV with 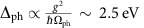 (Methods section). In other words, the electron-lattice interaction is responsible for half of the effective CT energy in LCO. To stress this point, Fig. 2c shows results obtained from a similar model where the e-ph interaction is taken out of the analysis. To even qualitatively reproduce the positions of the ZRS and CT excitations in this electronic-only model, Δ_el_ must now be increased to 4.6 eV. Furthermore, the phonon satellite peaks are absent in the quasi-elastic and *dd* excitation regions. This is a clear deficiency in the electronic-only model that is corrected only when the e-ph interaction is included. It is clear that the electron-lattice coupling plays a very significant role in establishing the effective value of Δ in LCO.

We note that there is a small discrepancy between the theory and experiment; namely, the relative intensity of the observed second phonon line with respect to the first one is slightly stronger than the one captured by the cluster calculation. While increasing the value of *g* does increase the intensity of the second phonon excitation relative to the first^18^, the single-mode model we have adopted always produces a diminishing intensity in successive phonon excitations. (We have also examined nonlinear e-ph interactions but these are unable to account for this discrepancy.) We therefore speculate that increased intensity in the second phonon excitation is due to multi-phonon processes that cannot be included in our calculations due to the necessary truncation of the phonon Hilbert space (Methods section). For these reasons, we selected *g*=0.2 eV, which is consistent with the Madelung energy considerations. This value also provides a conservative estimate for the lattice contribution to Δ.

**Figure 3:**
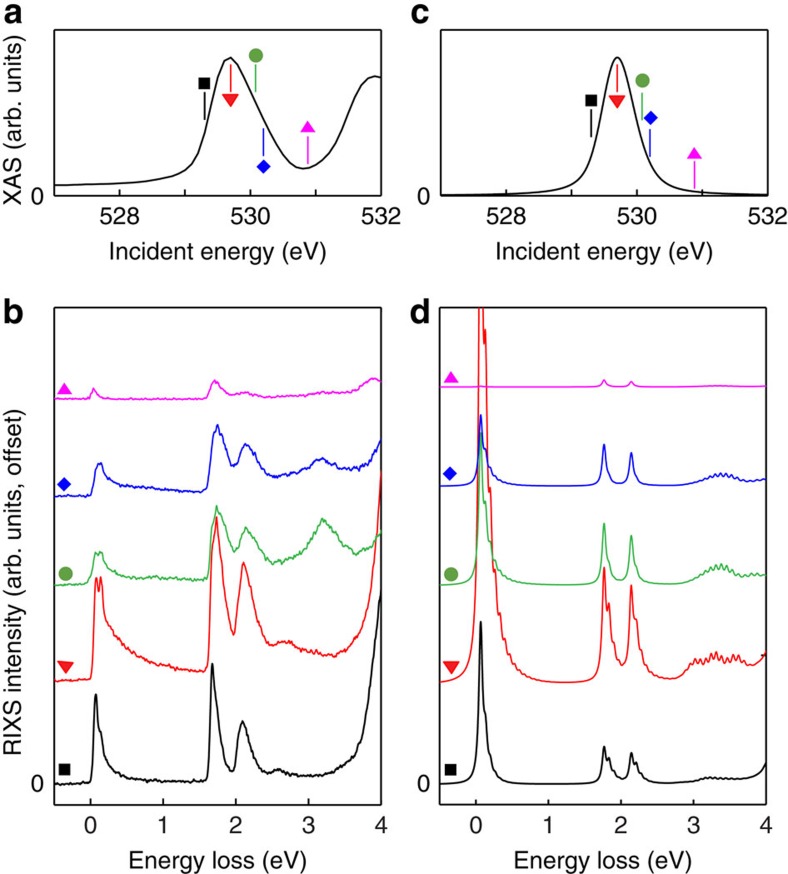
The incident photon energy dependence of the RIXS spectra. (**a**) and (**c**) shows the measured and calculated XAS spectra, respectively. Calculations were performed using the model including coupling to the lattice. The measured and calculated RIXS spectra as a function of the incident photon energy are shown in (**b**) and (**d**), respectively. The RIXS spectra have been offset for clarity and the incident photon energy is indicated by the color-coded symbols in the corresponding XAS plots. The calculations have been broadened using a Gaussian line shape with a s.d. of 60 meV.

In Fig. 3 we compare the measured incident photon energy dependence to the predictions of the e-ph coupled model as an additional verification. Here a resonance behaviour in the experimental data is observed, where the phonon excitations emanating from the elastic line and *dd* excitations persist to higher energy losses as *ℏ*Ω_in_ resonates with the upper Hubbard band excitation in the XAS. Our experimental observations are in agreement with prior O K-edge measurements on the related CYCO system^18^. (In both materials, the observed resonance behaviour is damped with respect to similar behaviour observed in gas-phase oxygen molecules^33^. This is due to the increased number of core-hole decay channels present in the solid^18^.) Our model with the e-ph interaction reproduces these features well. In contrast, the electronic model without the e-ph coupling fails to capture these features. This underscores once more the importance of the e-ph interaction for understanding the RIXS spectra on even the qualitative level.

## Discussion

We have performed oxygen K-edge RIXS measurements on the edge-shared one-dimensional cuprate LCO, revealing clear phonon excitations in the RIXS spectra. These excitations are well captured by a model that includes coupling to a Cu–O bond-stretching optical phonon mode, which modulates the on-site energy of the Cu orbitals and leads to a substantial renormalization of the effective CT energy. This renormalization is not a simple effect related to the formation of the core hole. The non-zero e-ph interaction that we infer here is present in the system regardless of the existence of the core hole. Thus the corresponding renormalization of the CT energy will also be present in other spectroscopies such as optical conductivity (Supplementary Fig. 1)^22^,^30^, EELS^31^ and inelastic neutron scattering^32^ (Supplementary Note 1).

Our results show that the e-ph interaction is of relevance to the Zaanen–Sawatzky–Allen classification of this material, where the lattice contribution to the CT energy accounts for nearly half of the total value. Since the ensuing renormalizations can be very large in materials possessing substantial e-ph couplings, we expect that such considerations will prove to be important in other families of quasi-one-dimensional correlated systems, where the lattice motion cannot be effectively screened. For example, the related spin-chain system CYCO likely has a large lattice contribution to the CT energy.

## Methods

### Sample preparation

LCO samples were grown under elevated gas pressure (in a gas mixture of Ar:O_2_ with a ratio of 4:1 at the total pressure of 50 bar) in a vertical travelling solvent-floating zone facility with optical heating^34^. The powder for the feed rods of LCO was prepared by grinding and sintering LiOH (Isotec, 99.9% of ^7^LiOH powder was used) and CuO (Chempur 99.99%) at 750 °C. Because the powder was single phase after the first sintering, no further annealing was done to avoid vaporization of lithium. The single-phase powder was pressed to polycrystalline rods (EPSI Engineered Pressure Systems; 3,500 bar) in latex tubes and sintered again at 800 °C for 34 h.

### RIXS measurements

The RIXS experiments were performed at the ADRESS beamline of the Swiss Light Source, Paul Scherrer Institut, using the SAXES spectrometer^35^,^36^. All spectra were recorded with *σ*-polarized light in the scattering geometry shown in Fig. 1a (the scattering angle was 130°, with an incidence angle of 65°). No momentum was transferred into the system along the direction of the chain using this geometry. The combined energy resolution was between 50 and 60 meV at the oxygen K-edge (*ℏω*_in_∼530 eV). About 150 photons were collected on the *dd* excitations (maximum intensity) during 2 h of data acquisition at an energy resolution of 60 meV (RIXS spectra of Fig. 2a). About 300 photons were collected on the *dd* excitations (maximum intensity) during 8 h of data acquisition at an energy resolution of 50 meV (RIXS spectra of Fig. 2d,f). The samples were cleaved *in situ* at a pressure of ∼5 × 10^−10^ mbar and a temperature *T*=20 K. The surface of the crystal was perpendicular to the [101] axis such that the CuO_4_ plaquettes were tilted 21° from the surface.

### XAS and RIXS intensities

The RIXS spectra at the oxygen (O) K-edge (1*s*→2*p*) were calculated using the Kramers–Heisenberg formula^5^,^6^,^37^. If the incoming and outgoing photons have energies (polarizations) *ℏω*_in_ (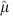) and *ℏω*_out_ (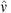), respectively, then the RIXS intensity is given by


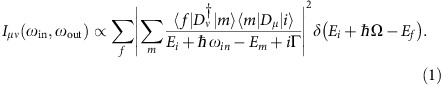


Here, *ℏ*Ω=*ℏω*_out_−*ℏω*_in_ is the energy loss; |*i*〉, |*m*〉 and |*f*〉 denote the initial, intermediate and final states of the RIXS process, respectively, with eigenenergies *E*_*i*_, *E*_*m*_ and *E*_*f*_, respectively; and Γ is the lifetime of the core-hole, which we assume is independent of the intermediate state.

The 1*s*→2*p* transition is induced by the dipole operator *D*_*μ*_. If no momentum is transferred to the sample (**q**=0) by the incoming photon, then the dipole operator is given by


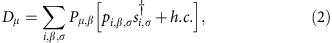


where 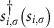 creates (annihilates) a 1*s* core-hole of spin *σ* on O site *i*, 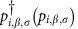 creates (annihilates) a spin *σ* hole in the O 2*p*_*β*_ orbital on the same site and 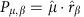 is the projection of the photon polarization onto the orientation of the O 2p_*β*_ orbital. For the scattering geometry shown in Fig. 1a, the transition operators are


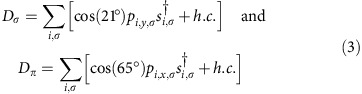


for *σ* and *π*-polarized light, respectively. (Note that the *p*_*z*_ orbitals do not appear in these operators since we do not include them in our Hilbert space, see below.) Since the polarization of the outgoing photon was not measured in the experiment, the total intensity is given by an incoherent sum over outgoing polarizations *I*_*σ*_=∑_*μ*_ *I*_*μ*,*σ*_ and *I*_*π*_=∑_*μ*_ *I*_*μ*,*π*_. Here, the reader should not confuse the polarization index σ with the spin index. In the main text we show results calculations *I*_*σ*_ polarization.

### Model Hamiltonian

The eigenstates |*i*〉, |*m*〉 and |*f*〉 were obtained from exact diagonalization of a small Cu_3_O_8_ cluster with an edge-shared geometry and open boundary conditions, as shown in Fig. 1b. The orbital basis contains the 3*d*_*xy*_, 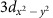 and 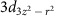 orbitals on each Cu site, and the O 2*p*_*x*,*y*_ orbitals on each O site. Throughout, *α* and *α*^′^ are used to index Cu orbitals, *β* and *β*′ are used to index O orbitals and the roman indices *i*, *j* index the lattice sites.

The full Hamiltonian is *H*=*H*_0_+*H*_e−e_+*H*_ph_+*H*_e−ph_, where *H*_0_ and *H*_ph_ contain the non-interacting terms for the electronic and lattice degrees of freedom, respectively, *H*_e−e_ contains the electron–electron interactions, and *H*_e−ph_ contains the e-ph interactions.

The non-interacting terms for electronic degrees of freedom are


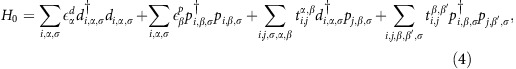


where the Cu operators 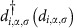 and O operators 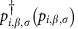 create (annihilate) a hole of spin *σ* in orbital *α* (or *β*) on atomic site *i*. In Equation (4) 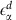 and 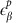 are the on-site energies of the Cu and O orbitals, respectively, while 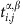 and 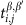 are the Cu–O and O–O hopping integrals, respectively.

The electron–electron interactions include the on-site inter- and intra-orbital interactions on each Cu 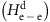 and O 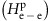 site, the nearest-neighbor Cu–O repulsion and exchange interactions 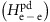, and the nearest-neighbor Cu–Cu repulsion 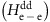. The Cu on-site interactions take the form


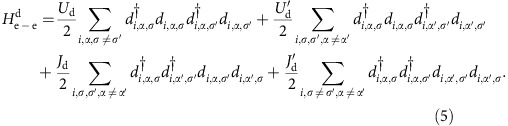


The form of on-site O interactions, 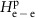, is the same. The nearest-neighbour Cu–O interactions take a similar form 
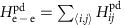
, where the sum is over nearest-neighbor Cu and O sites and


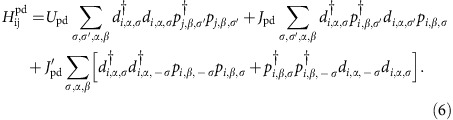


Finally, the Cu–Cu nearest-neighbor repulsion is given by


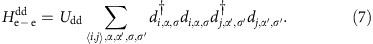


For the lattice model *H*_ph_ and *H*_e−ph_, we considered a single-oxygen Cu–O bond-stretching mode that compresses the Cu–O bond in the direction perpendicular to the chain direction, as indicated by the arrows in Fig. 1b. The reduction to a single-phonon mode is required to maintain a manageable Hilbert space for the problem; however, this approximation is sufficient to describe the phonons in the related system CYCO (ref. 18). In principle, these bond-stretching phonons couple to the carriers in the chain via two microscopic mechanisms: the first is via the direct modulation of the interchain hopping integrals. The second is via a modification of the Cu site energies. The magnitude of the former can be estimated from the distance dependence of the atomic hopping parameters. The magnitude of the latter can be estimated using an electrostatic point charge model for the Madelung energies^2^,^22^. We carried out such calculations using known structural data^20^ and obtained the distance dependence of 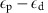 (neglecting crystal field effects) for static compressions of the CuO_2_ chain, as shown in Fig. 1b. The results are shown in Fig. 1c, where we obtain an e-ph coupling strength *g*∼0.24 eV. Calculations were then carried out for both coupling mechanisms and the Cu site energy modulation was found to have the the largest impact on the calculated RIXS spectra. We therefore neglected the modulation of the hopping integrals here for simplicity and introduced a Holstein-like coupling to the Cu site energies. Within this model the Hamiltonian for the lattice degrees of freedom is


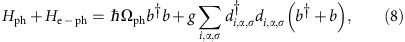


where *b*^†^ (*b*) creates (annihilates) a phonon quanta of the compression mode. The hilbert space for the lattice degrees of freedom is truncated at a large number of allowed phonon quanta (∼200). We have checked to ensure that our results are not significantly changed for further increases in this cut-off.

Finally, when calculating the intermediate states in Equation (1), the Hamiltonian is augmented with the appropriate terms describing the Coulomb interaction with the core-hole^8^. Specifically, we add


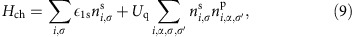


where 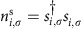 is the number operator for the 1*s* core level on oxygen site *i*, 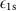 is the energy of the O 1*s* core-hole and *U*_q_ is the core-hole potential.

### Model parameters

The multi-band Hamiltonian has a number of parameters that can be adjusted; however, we are constrained by multiple experimental probes. To this end we have a well-established set given in ref. 9, which simultaneously reproduces high-energy features in the RIXS data^9^, Cu–Cu exchange interactions inferred from inelastic neutron scattering measurements^32^, and optical conductivity and EELS measurements^31^ in LCO. Given this level of descriptive power, we adopt the same parameter set here.

When the e-ph interaction is included in the calculation we take (in units of eV) 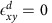, 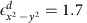, 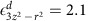, 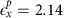 and 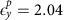. The Cu–O hopping integrals are (in eV) 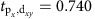, 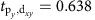, 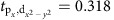, 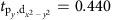, 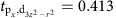, and 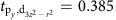. The O–O hopping integrals are (in eV) 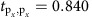 (0.240) and 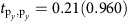, for hopping parallel (perpendicular) to the chain direction. The Hubbard and Hunds interactions for the Cu sites are given by the Racah parameterization^38^ with *A*=6.45, *B*=0.25 and *C*=0.35. The oxygen interactions are *U*_p_=4.1, 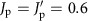 and 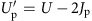. The extended interactions are *U*_dd_=0.4, *U*_pd_=0.8, 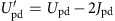 and *J*_pd_=0.096. The phonon energy is taken to be *ℏ*Ω_ph_=74 meV, and the e-ph coupling strength *g* is taken as a variable. The core-hole parameters are *U*_q_=4.3 eV and Γ=150 meV for the oxygen K-edge.

All of the parameters remain the same for the calculations performed without e-ph coupling with the exception of Γ=300 meV, 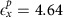, and 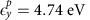. It should be noted that this parameter set assumes a larger value for the CT energy in comparison to ref. 9, and fails to capture the phonon features in the RIXS data (Fig. 2c). To correct this, we take the bare CT energy 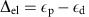 and the bare e-ph interaction strength *g* as fitting parameters and keep all other model parameters to be the same as those listed above when the e-ph interaction is included. We therefore regard the CT energy Δ used in ref. 9 as an effective CT energy, which includes the effects of the e-ph interaction.

### Madelung energies

The coupling to the phonon mode enters into our calculations to first order in displacement via the modulation of the Cu and O Madelung energies *V*_Cu_ and *V*_O_, respectively. The Madelung energy for a given site *i* can be estimated using an ionic model, and is given by 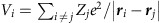, where *Z*_*j*_*e* is the formal charge associated with the atom at site *j*. Neglecting crystal field effects, the difference between the Cu and O site energies is related to the difference in Madelung energies Δ*V*_M_=*V*_O_−*V*_Cu_ by^2^


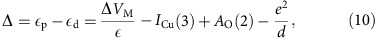


where *A*_O_(2) is the second electron affinity of oxygen, *I*_Cu_(3) is the third ionization energy of Cu, *d* is the Cu–O distance and 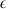 is the high-frequency dielectric constant. The distance dependence of Δ can be estimated by calculating Δ*V*_M_ using the Ewald summation technique and the known structural data^20^. Assuming 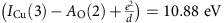 and 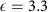, we arrive at Δ=3.2 eV for the experimental lattice parameters, in agreement with ref. 22. This value, however, is substantially lower than the value inferred from our RIXS study if the e-ph interaction is excluded.

To estimate the strength of the e-ph interaction, we performed calculations where the Cu–O plaquettes were compressed by a distance *u* in the directions indicated by the arrows in Fig. 1b. The resulting distance dependence of Δ(*u*) is plotted in Fig. 1c, where a linear dependence of Δ occurs over a wide range of displacements. To capture this, we parameterize the Cu site energy as 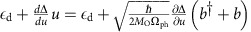, where *M*_O_ is the mass of oxygen. This results in an e-ph coupling of the form given in equation (8) with 
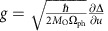
. A linear fit to Δ(*u*) (shown in Fig. 1c) gives 
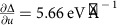
, which yields *g*∼0.24 eV. It should be stressed that this value of *g* is an estimate based on a point charge model, however, it gives us an idea of the expected coupling strength.

### Renormalization of the charge-transfer energy

As discussed in the main text, in the ground state of the LCO chain the oxygen atoms will shift to new equilibrium positions in response to the linear e-ph coupling terms of the Hamiltonian. This situation can be qualitatively understood by introducing shifted phonon operators 
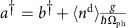
 and 
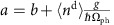
, where 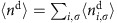 is the average number of holes on the Cu site in the ground state. These new operators yield a shifted atomic position given by 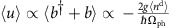. This shift of position is responsible for the renormalization of the CT energy. After this transformation is made the phonon and e-ph coupled terms of the Hamiltonian (equation (8)) reduce to


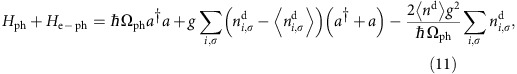


where we have dropped an overall constant. The second term describes the coupling to the lattice in the new equilibrium position, which is proportional to the fluctuation in Cu charge density from its ground state value. The third term can be folded into the definition of the Cu site energy with 
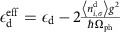
. This gives an effective CT energy Δ_eff_=Δ_el_+Δ_ph_ where 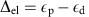 and 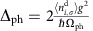. From these considerations one can also see that no isotope effect is predicted for Δ_ph_, since both *g*^2^ and *ℏ*Ω_ph_ are proportional to the inverse of the mass of oxygen.

## Additional information

**How to cite this article:** Johnston, S. *et al*. Electron-lattice interactions strongly renormalize the charge-transfer energy in the spin-chain cuprate Li_2_CuO_2_. *Nat. Commun.* 7:10563 doi: 10.1038/ncomms10563 (2016).

## Supplementary Material

Supplementary Information Supplementary Figure 1, Supplementary Note 1 and Supplementary References
