# Supplementary material for: Electron-lattice interactions strongly renormalize the charge-transfer energy in the spin-chain cuprate Li2CuO2
Source: Nat Commun. 2016 Feb 17;7:10563. doi: 10.1038/ncomms10563 (PMC4757783; doi:10.1038/ncomms10563)
Supplement: Supplementary Information — Supplementary Figure 1, Supplementary Note 1 and Supplementary References [file ncomms10563-s1.pdf]

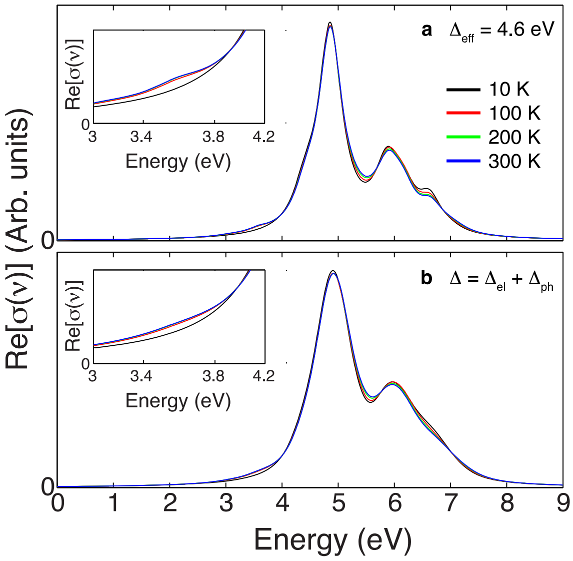

Supplementary Figure 1: Calculated real part of the optical conductivity. Panels **a** and **b** show results for the effective electronic model and the electronic plus electron-phonon coupled model used in the main text. The inset shows a zoom-in of the optical conductivity in the energy range 3 – 4.2 eV, where a weak Zhang-Rice singlet excitation is observed.

Supplementary Note 1:

Our model provides a good description of the RIXS data. It also provides reasonable descriptions of both the optical conductivity and the exchange interactions measured experimentally. For example, fitting the spin wave dispersions obtained from inelastic neutron scattering experiments with linear J_1-J_2 spin-wave theory produces estimates for the nearest- and next-nearest-neighbor Cu-Cu exchange interactions of $J_1 = -19 \pm 0.4$ meV and $J_2 = 6.2 \pm 0.4$ meV, respectively [1]. These values can be extracted from the lowest lying eigenstates in our Cu_3O_8 cluster calculations [2]. Our model with the electron-phonon (e-ph) interactions gives (in units of meV) $J_1 = -17$ and $J_2 = 3.5$. Our effective model without the e-ph interaction gives $J_1 = -17$ meV and $J_2 = 3.2$ meV. Both sets of results are in reasonable agreement with the experimental values.

We can also calculate the optical conductivity from our two models. The method for computing the real part of the optical conductivity $\mathrm{Re}[\sigma(\nu)]$ at finite temperatures is given in Ref. 4 and this quantity can be readily obtained from our ED calculations. The results are shown in Supplementary Fig. 1, where Supplementary Fig 1a shows the results for the effective electronic model and Supplementary Fig 1b shows results for the model with the inclusion of the e-ph interaction. In both cases, a large peak is observed in $\mathrm{Re}[\sigma(\nu)]$ for $\nu \sim 4 - 5$ eV, which is consistent with experiments [3, 4]. (In this case we slightly overestimate the peak position due to finite size effects associated with the use of the Cu_3O_8 cluster [4].)

The fact that both the electronic and electronic plus lattice models produce peaks at the same energies underscores several points. First, the e-ph coupling we infer in our RIXS study is not simply a core hole effect. Rather, it reflects the coupling present in the material even before the core-hole is created during the RIXS process, since neither INS nor optical conductivity measurements create a core hole. Second, the peak position in the optical conductivity and the effective Heisenberg interactions are sensitive to the total effective charge transfer energy, and not the electronic-only contribution. And third, our model derived for the RIXS spectra captures well the other spectroscopic data and therefore our conclusions are not limited to describing the RIXS experiment.

Supplementary references:

1. W. E. A. Lorenz, R. O. Kuzian, S.-L. Drechsler, W.-D. Stein, N. Wizent, G. Behr, J. Málek, U. Nitzsche, H. Rosner, A. Hiess, W. Schmidt, R. Klingeler, M. Loewenhaupt, and B. Büchner, *Highly dispersive spin excitations in the chain cuprate Li_2CuO_2*. Europhys. Lett. **88**, 37002 (2009).
2. C. Monney, V. Bisogni, K.-J. Zhou, R. Kraus, V. N. Strocov, G. Behr, J. Málek, R. Kuzian, S.-L. Drechsler, S. Johnston, A. Revcolevschi, B. Büchner, H. M. Rønnow, J. van den Brink, J. Geck, and T. Schmitt, *Determining the Short-Range Spin Correlations in the Spin-Chain Li_2CuO_2 and $CuGeO_3$ Compounds Using Resonant Inelastic X-Ray Scattering.* Phys. Rev. Lett. **110**, 087403 (2013).
3. Y. Mizuno, T. Tohyama, S. Maekawa, T. Osafune, N. Motoyama, H. Eisaki, and S. Uchida, *Electronic states and magnetic properties of edge-sharing Cu-O chains*. Phys. Rev. B **57**, 5326–5335 (1998).
4. J. Málek, S.-L. Drechsler, U. Nitzsche, H. Rosner, and H. Eschrig, *Temperature-dependent optical conductivity of undoped cuprates with weak exchange*. Phys. Rev. B **78**, 060508(R) (2008).
